# Sparrow Modeling to Understand Water-Quality Conditions in Major Regions of the United States: A Featured Collection Introduction^1^

**DOI:** 10.1111/j.1752-1688.2011.00585.x

**Published:** 2011-10

**Authors:** Stephen D Preston, Richard B Alexander, David M Wolock

## Background

Management of the quality of the Nation's water requires large quantities of information describing current conditions and related environmental factors. Such information is expensive to collect and difficult to interpret over large scales. To support the efficient use and interpretation of available water-resource information, the U.S. Geological Survey (USGS) developed a spatial water-quality modeling framework known as SPAtially Referenced Regressions On Watershed attributes or SPARROW (Smith *et al.*, 1997). SPARROW is a hybrid empirical/process-based mass-balance model that can be used to estimate the major sources and environmental factors that affect the long-term supply, transport, and fate of contaminants in streams. The spatially explicit model structure is defined by a river reach network coupled with contributing catchments. The model is calibrated by statistically relating watershed sources and transport-related properties to monitoring-based water-quality load estimates. The model results can inform scientific understanding and management by providing a tool for identifying sources of constituents that affect water quality over a wide range of spatial scales, providing estimates of mass contributions from sources to streams and downstream receiving waters. Further details on SPARROW are available at: http://water.usgs.gov/nawqa/sparrow/.

SPARROW models have been previously developed in the United States (U.S.) over spatial extents ranging from the conterminous U.S. (Smith *et al.*, 1997; Alexander *et al.*, 2000, 2008) to large regions such as the Chesapeake Bay watershed (Preston and Brakebill, 1999) and smaller watersheds such as those draining to the North Carolina coast (McMahon *et al.*, 2003). SPARROW models have been applied in many ways to improve the understanding of water-quality conditions and controlling factors, including: (1) identifying major sources of nutrients in streams of the conterminous U.S. (Smith *et al.*, 1997; Alexander *et al.*, 2008) and in individual watersheds in support of Total Maximum Daily Load (TMDL) assessments (McMahon *et al.*, 2003; Moore *et al.*, 2004), (2) understanding the role of stream processing in the delivery of nutrients to coastal waters, such as the Gulf of Mexico (Alexander *et al.*, 2000, 2008), (3) identifying the sources of salinity affecting water supply in the southwest (Anning *et al.*, 2007), and (4) understanding the environmental factors affecting sediment loading to the Chesapeake Bay (Brakebill *et al.*, 2010). SPARROW models have also been applied in New Zealand (Alexander *et al.*, 2002) and are now being developed for evaluating water-quality conditions in other parts of the world.

**FIGURE 1:**
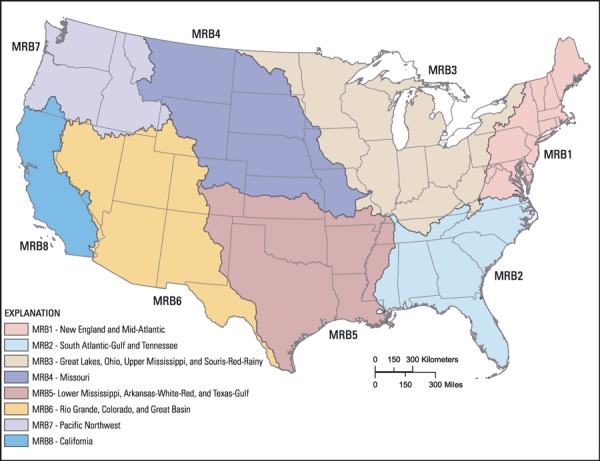
U.S. Geological Survey National Water Quality Assessment (NAWQA) Program Major River Basins (MRB) of the conterminus United States.

Recent efforts by the USGS include a systematic approach to develop regional models of stream water quality for the conterminous U.S. Descriptions of the efforts to build such models, and their results and management implications, are the focus of this Featured Collection of articles in the *Journal of the American Water Resources Association*. The models described in this collection were developed as part of the USGS National Water Quality Assessment (NAWQA) program efforts to assess the water quality of the Nation's streams. NAWQA adopted SPARROW as a modeling framework that could be used to define the spatial distribution of water-quality conditions and identify the primary environmental factors that affect water quality regionally. The regions that form the spatial domains of the models are designated as “Major River Basins” (MRBs) (Figure 1). Eight MRBs are defined for the conterminous U.S. SPARROW models have been developed for seven of those regions (MRB1 through MRB7) (Preston *et al.*, 2009) and models for MRB8 (California) are currently under development. Separate models were developed for each region to provide a regional focus on environmental conditions, data compilation, and management issues. Nutrients (total nitrogen, total phosphorus) are the focus of modeling in all regions except MRB6 – the arid southwest, where dissolved solids were modeled to address the regionally important issue of salinity.

## Overview of the Featured Collection

The Featured Collection begins with a synthesis by Preston *et al.* (this issue) of the results of the six regional modeling studies of stream nutrients (total nitrogen, total phosphorus). The authors provide a post analysis of the regional SPARROW models to assess their consistency as to the major sources and environmental factors that explain spatial patterns in mean annual nutrient loads across regions of the conterminous U.S. The results indicate the nearly universal importance of urban and agricultural sources of nutrients, but highlight regional differences in the nutrient contributions to streams from diffuse urban sources, agricultural sources (fertilizer, animal manure), and specific background sources and environmental processes (e.g., soil phosphorus, nitrogen fixation in forests, channel erosion).

This is followed by three articles that describe the methods for compiling several key geospatial datasets that are used to develop the regional models. These geospatial datasets were developed at a national scale and applied to the regions to ensure consistency in the methodologies; in selected regions, data were developed to describe specific nutrient sources that are important for those areas (e.g., Wise and Johnson, this issue). Brakebill *et al.* (this issue) provide an overview of digital stream networks that define the spatial framework of SPARROW models. That article documents the background of some widely used national stream network datasets and documents the river network and associated attributes that are used as the infrastructure for the regional SPARROW models. Saad *et al.* (this issue) describe the compilation of stream monitoring data from nearly 3,000 sites in the U.S., and the techniques used to derive estimates of the long-term mean annual nutrient loads that are used to calibrate the regional SPARROW models. The article describes methods used, describes results of the database development efforts, and provides information about the availability of water-quality and water-quantity information both regionally and nationally. Maupin and Ivahnenko (this issue) describe the effort to develop nationally consistent estimates of municipal and industrial wastewater discharges of nitrogen and phosphorus. Such information is generally available through the U.S. Environmental Protection Agency's (USEPA) Permit Compliance System (PCS) database. However, use of the PCS database requires additional processing to validate discharge values and facility locations and other reported information. This article describes the methods required to develop a national-scale point source database for use in the regional SPARROW models and the results of the effort and ramifications for spatial modeling.

The regional models are described in seven articles, each of which provide the results of the models and an example management-relevant application for a water-quality issue specific to that region. Moore *et al.* (this issue) describe their modeling results for the northeast (MRB1). They provide estimates of nitrogen loading from selected major tributaries to estuaries and identify lakes that are likely to be impacted by phosphorus loads. García *et al.* (this issue) describe nutrient sources and transport in streams of the southeast (MRB2). In addition to the importance of agricultural and urban sources, they identify naturally occurring phosphorus associated with soil-parent rock as a regionally important source of phosphorus in streams of the southeast. Robertson and Saad (this issue) describe their modeling results for the upper Midwest (MRB3) and provide an assessment of loading to the Great Lakes system from U.S. drainages, including the identification of the most important contributing areas and types of sources. On the basis of their work, agriculture and point sources (municipal and industrial wastewaters) were the largest sources of nutrients to the Great Lakes, with the watersheds draining to Lake Erie having the highest areal nutrient loadings among all watersheds in the Great Lakes region. Brown *et al.* (this issue) describe their model for the Missouri River drainage (MRB4) and highlight the important effects of irrigation and reservoirs on the water quality of streams in that region. They find nitrogen loads leaving the Missouri drainage may be reduced by denitrification in the soils associated with irrigated lands, and that nutrient losses in reservoirs account for appreciable reductions in nitrogen and phosphorus loads. Rebich *et al.* (this issue) describe their model for the lower Midwest (MRB5) and provide an evaluation of nutrient loading from the region as a whole as well as to local embayments along the Gulf of Mexico coast. Atmospheric deposition was found to be the largest source of nitrogen in that region and agricultural fertilizer was found to be the largest source of phosphorus. Anning (this issue) describes a model of salinity in the southwestern part of the country (MRB6), which is used to identify the primary sources of salinity to streams, and assess the relative importance of natural geologic materials and human activities related to agricultural water use. Anning finds that although the areal loadings of dissolved solids to streams (load per unit area) from agricultural lands are more than an order of magnitude higher than the loadings from geologic materials, the relative contributions of dissolved solids to streams, on average, from natural geologic materials and agricultural sources are nearly equal across the region. The author describes the utility of the model for developing more effective salinity control strategies in southwestern watersheds. Lastly, Wise and Johnson (this issue) describe their model of the Pacific Northwest (MRB7). They provide information on both natural (e.g., nitrogen fixation in forests) and anthropogenic sources of nutrients to streams, and report the fraction of streams within watersheds that are estimated to have nutrient concentrations that exceed the recommended USEPA nutrient criteria.

A rapidly growing need for the use of water-quality models is the capacity for water-resource managers to readily access the results and to explore the details of the model output without the assistance of model developers and technical experts or the need for special software or training. Booth *et al.* (this issue) describe a new innovative approach for providing this capacity through a web-based decision support system (DSS). The DSS allows users to access the regional SPARROW models that are described in the Featured Collection (and several prior SPARROW models), display the results from these models, or export the information for use in other applications. The system incorporates the spatial connectivity provided by the digital stream network that is inherent to SPARROW models and that provides a basis for evaluating the relations between upstream sources and downstream receiving waters. Furthermore, it allows users to modify model inputs based on potential scenarios of changes in watersheds that could result from management actions or other human activities. The DSS can be used with each of the regional SPARROW models (or previous national models) to explore and evaluate water-quality conditions in the conterminous U.S.

Finally, Schwarz *et al.* (this issue) describe a new technique for estimating regional variability in the coefficients of a national SPARROW model and illustrate the method using a previous national SPARROW model and its associated geospatial data for the conterminous U.S. The authors demonstrate that the simultaneous modeling of water-quality conditions across regions can provide more precise estimates of regional coefficients and model predictions than can be obtained through independently estimated regional models. The approach, which is complementary to the synthesis of the regional SPARROW models presented by Preston *et al.* (this issue), shows promise for future regional SPARROW modeling of water-quality conditions in the nation's streams.
